# Cryo-tomography and 3D Electron Diffraction Reveal the Polar Habit and Chiral Structure of the Malaria Pigment Crystal Hemozoin

**DOI:** 10.1021/acscentsci.4c00162

**Published:** 2024-07-04

**Authors:** Paul Benjamin Klar, David Geoffrey Waterman, Tim Gruene, Debakshi Mullick, Yun Song, James Boris Gilchrist, C. David Owen, Wen Wen, Idan Biran, Lothar Houben, Neta Regev-Rudzki, Ron Dzikowski, Noa Marom, Lukas Palatinus, Peijun Zhang, Leslie Leiserowitz, Michael Elbaum

**Affiliations:** † Faculty of Geosciences and MAPEX Center for Materials and Processes, University of Bremen, Klagenfurter Str. 2, 28359 Bremen, Germany; ‡ STFC, Rutherford Appleton Laboratory, Didcot OX11 0FA, U.K.; § CCP4, Research Complex at Harwell, Rutherford Appleton Laboratory, Didcot OX11 0FA, U.K.; ∥ Department of Inorganic Chemistry, Faculty of Chemistry, University of Vienna, Vienna 1090, Austria; ⊥ Department of Chemical and Biological Physics, Weizmann Institute of Science, 76100 Rehovot, Israel; # Diamond Light Source, Harwell Science and Innovation Campus, Didcot OX11 0DE, U.K.; 7 Department of Chemistry, Carnegie Mellon University, Pittsburgh, Pennsylvania 15213, United States; 8 Department of Molecular Chemistry and Materials Science, Weizmann Institute of Science, 76100 Rehovot, Israel; 9 Department of Chemical Research Support, Weizmann Institute of Science, 76100 Rehovot, Israel; 10 Department of Biomolecular Sciences, Weizmann Institute of Science, 76100 Rehovot, Israel; 11 Department of Microbiology and Molecular Genetics, Institute for Medical Research Israel-Canada, and The Kuvin Center for the Study of Infectious and Tropical Diseases, The Hebrew University-Hadassah Medical School, Jerusalem 9112010, Israel; 12 Department of Materials Science and Engineering, Carnegie Mellon University, Pittsburgh, Pennsylvania 15213, United States; 13 Institute of Physics of the Czech Academy of Sciences, Na Slovance 2, 182 21 Prague 8, Czechia; 14 Division of Structural Biology, Wellcome Trust Centre for Human Genetics, University of Oxford, Oxford OX3 7BN, U.K.

## Abstract

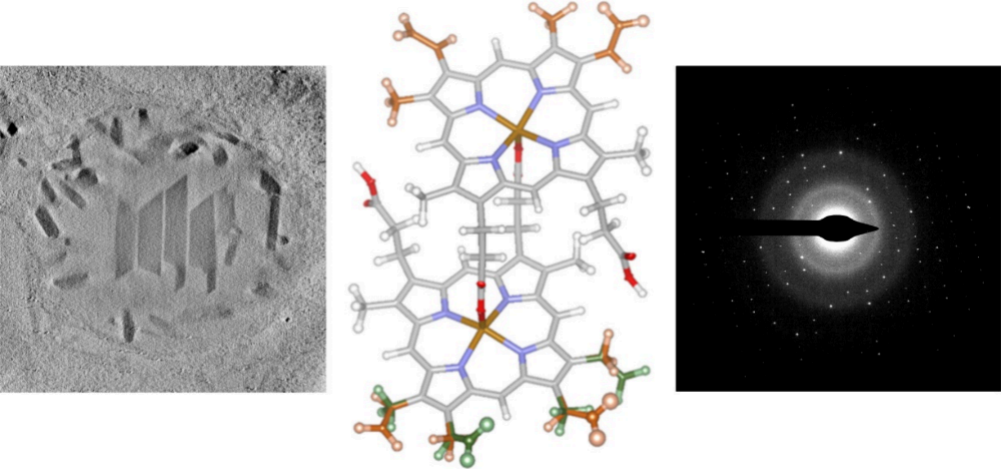

Detoxification of heme in *Plasmodium* depends on its crystallization into hemozoin. This pathway is a major target of antimalarial drugs. The crystalline structure of hemozoin was established by X-ray powder diffraction using a synthetic analog, β-hematin. Here, we apply emerging methods of *in situ* cryo-electron tomography and 3D electron diffraction to obtain a definitive structure of hemozoin directly from ruptured parasite cells. Biogenic hemozoin crystals take a striking polar morphology. Like β-hematin, the unit cell contains a heme dimer, which may form four distinct stereoisomers: two centrosymmetric and two chiral enantiomers. Diffraction analysis, supported by density functional theory analysis, reveals a selective mixture in the hemozoin lattice of one centrosymmetric and one chiral dimer. Absolute configuration has been determined by morphological analysis and confirmed by a novel method of exit-wave reconstruction from a focal series. Atomic disorder appears on specific facets asymmetrically, and the polar morphology can be understood in light of water binding. Structural modeling of the heme detoxification protein suggests a function as a chiral agent to bias the dimer formation in favor of rapid growth of a single crystalline phase. The refined structure of hemozoin should serve as a guide to new drug development.

## Abstract 2

The polar shape of hemozoin crystals reflects the isomeric composition of the heme dimer in the unit cell.

## Introduction

Malaria is a deadly disease that remains endemic to much of the world despite extraordinary efforts to its eradication.^1^*Anopheles* mosquitos are the universal vector, and several species of *Plasmodium* affect humans, where they infect first the liver and then the red blood cells. Blood cell cytoplasm, containing primarily hemoglobin, is taken up into the parasite and delivered to the acidified digestive vacuole. There, the hemoglobin protein is catabolized by falcipain 2 and other enzymes as a source of biomolecules for parasite growth and multiplication. Hemoglobin is a tetrameric protein, each unit of which binds a single heme monomer via interaction of the central iron ion with a nearby histidine residue. The heme released by proteolysis is toxic to the parasite, however. Detoxification of the heme involves its sequestration into physiologically insoluble crystals of hemozoin, also known diagnostically and historically as the malaria pigment. Disease symptoms include anemia from loss of hemoglobin and severe paroxysms resulting from immune response to hemozoin released to the bloodstream.^2^

Growth of hemozoin is a target of common antimalarial drugs,^3−6^ leading to death of *Plasmodium* cells upon overload of free heme. Proposed mechanisms of crystal growth inhibition include, on one hand, the formation of adducts with the exposed heme such that their incorporation into the hemozoin crystal is prevented, and, on the other hand, interaction of the drug molecule with an incipient or growing facet such that further crystal growth is slowed. (A classic example of the latter are antifreeze proteins for water ice.^7 ,8^) Atomic force microscopy studies using the synthetic analog of hemozoin, β-hematin, have explored the nature and consequence of drug binding to specific crystal faces *in vitro*.^9,10^ Interaction of chloroquine with hemozoin *in situ*, within the parasite, has been suggested by imaging of a fluorescent derivative of the drug,^11^ and more directly in a recent study using synchrotron-based correlative imaging and spectroscopic methods to detect the close analog bromoquine.^3^ Intriguingly, both studies found the drug in association with the digestive vacuole inner membrane as well as other cellular membranes. Effective adsorption of a drug to the crystal surface depends on specific chemical interaction. Therefore, a precise determination of the crystal structure and morphology, in particular of its exposed surfaces, is of prime medical importance as well as fundamental interest.

A landmark study by X-ray powder diffraction (XRPD) revealed the crystalline structure of β-hematin, and pointed out its similarities to the biogenic hemozoin.^12^ The unit cell contains not the monomeric heme present in blood hemoglobin, but rather a cyclic dimer. The structure was refined in a triclinic, centrosymmetric (space group *P*1̅) arrangement with one dimer per unit cell. Cyclic H-bonds between propionic acid groups form chains linking the hematin dimers along the ***a***–***c*** direction. Synthetic β-hematin is commonly grown in low-polarity solvent due to the poor solubility of heme in water. The growth morphology is rod-like with well-developed {100} faces and {010} side faces of variable width, capped by slanted {011} end faces. These indices have been determined by transmission electron microscopy and diffraction,^13^ and are in agreement with a theoretical growth form based on attachment energies.^14^

**Figure 1:**
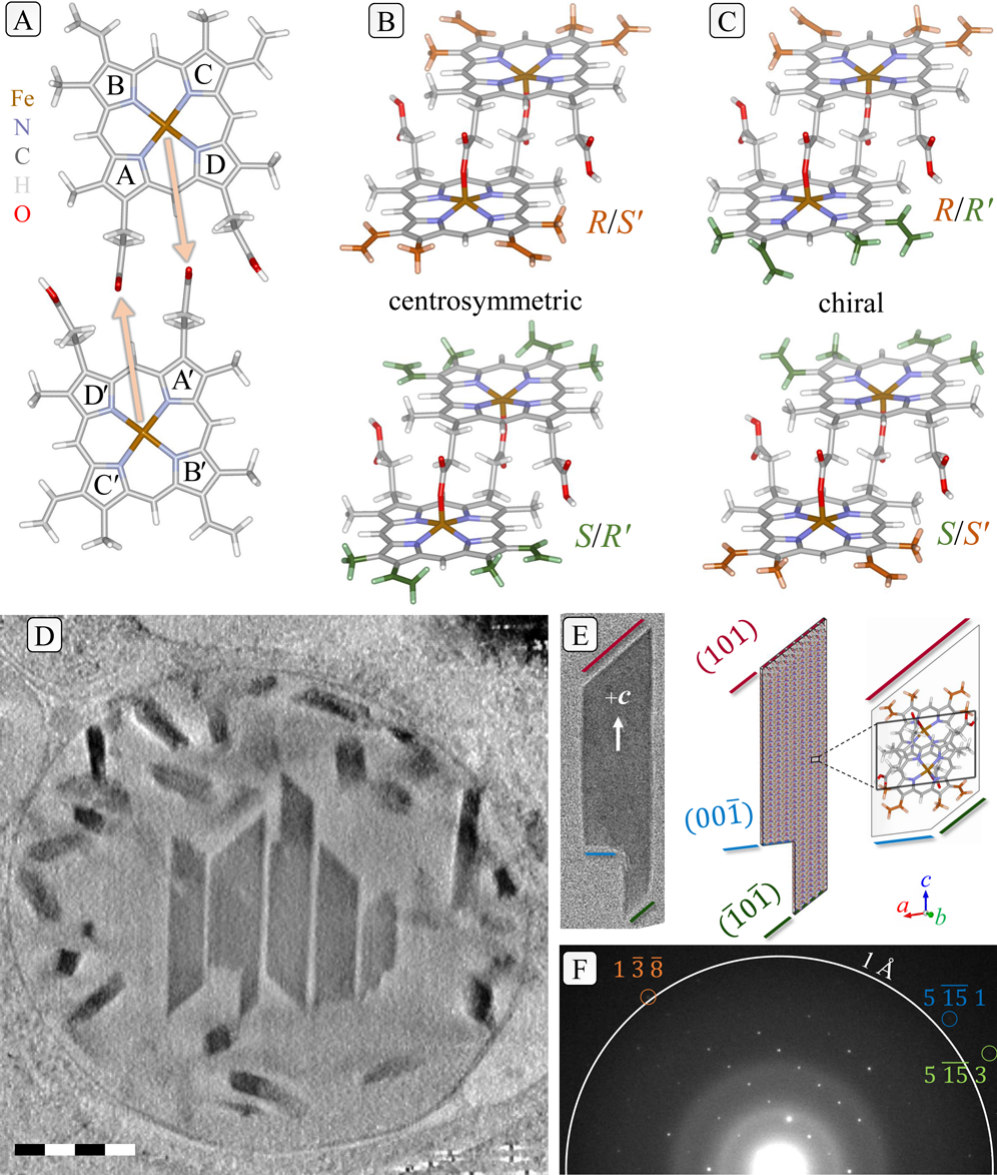
Isomers of the unit cell and polar crystal morphology. (A). Prior to crystallization, two heme monomers interlink to form cyclic hematin dimers via the binding of each central Fe ion to one of the two propionic acid tails (pyrrole ring labels included). The pro-chiral nature of the heme supports formation of four distinct stereoisomers. (B). Centrosymmetric *R/S′* and *S/R′* dimers differ in binding of the Fe to the propionate moiety at the A or D pyrrole. (C). *R/R′* and *S/S′* are enantiomers. Colors of methyl and vinyl groups indicate the symmetrical relationship: both sides either orange or green for centro-symmetry and mixed for chiral dimers. See also Supplementary Figure S3. (D). Thick virtual section from a tomographic reconstruction of an intact digestive vacuole showing large crystals in the center and numerous smaller crystals decorating the periphery. Note the polar shapes of the crystals. The complete reconstruction appears in Supplementary Movie S1. Scale bar 500 nm. (E) An exemplary crystal used in the diffraction study, with annotation of faces by Miller indices. (F) An exemplary diffraction pattern showing sharp spots reaching resolution beyond 1 Å.

The pro-chiral nature of the heme supports four configurations of the constituent dimer.^14−17^ Specifically, methyl–vinyl pairs opposite the propionic acids are arranged asymmetrically, defining *re* (*R*, rectus) and *si* (*S*, sinister) heme faces. Hemoglobin recognizes the asymmetry, so that oxygen binds the heme iron always on the *R* face; heme binding proteins in general distinguish the two inequivalent orientations.^18^ Cyclic dimers form distinct isomers depending on which faces are brought to contact: *R/S′*, *S/R′*, *R/R′*, or *S/S′*. The first two are centrosymmetric, whereas the latter two are chiral enantiomers with pseudo-2-fold symmetry (Figure 1, Figure S3). In solution all four isomers may form, but including such a mixture in a lattice is likely to inhibit crystal growth as strains could be induced by clashes at the unit cell boundaries. Growth inhibition would of course be detrimental to parasite survival, which depends on rapid detoxification of the heme released from digested hemoglobin.

Straasø et al. interpreted XRPD data of synthetic hemozoin in terms of distinct major and minor centrosymmetric phases. The authors suggested that these phases represent *R/S′* and *S/R′* isomers, respectively, each containing additionally *R/R′* and *S/S′* dimers.^16,17^ (This is conceptually similar to crystallization from a racemic mixture into distinct crystals of *S* and *R* stereoisomers.). In contrast to the formation energy of isolated dimers, DFT calculations indicated a significant penalty to mix *R/S′* and *S/R′* isomers in the same lattice. Bohle et al. then reanalyzed the earlier powder diffraction and interpreted the data in terms of a disordered structure of *R/S′* and *S/R′* dimers in a 3:1 ratio.^19^ The authors, however, did not present figures of merit (e.g., *R* factors) as a function of the relative ratio. Moreover, it is not possible to distinguish between the *R/S′ + S/R′* mixture and the mixture *R/R′ + S/S′*, and, in addition, it would be difficult by powder diffraction to distinguish disorder in the hemozoin lattice from a mixture of different crystals with the same unit cell parameters. Complementary evidence that synthetic hemozoin is comprised of a mixture of the different isomeric hematin dimers also stemmed from a single-crystal X-ray diffraction study of a hematin dimer-DMSO solvate.^20^ More recently, a study of synthetic hemozoin by X-ray free electron laser diffraction^21^ suggested that the structure of nanocrystals may differ from that of larger ones due to conformational flexibility in the propionic chains. This difference might also reflect the *R/S′* and/or *S/R′* composition.^22^

Biogenic hemozoin grows in the complex environment of the parasite digestive vacuole (DV). Crystallization must be tightly coupled to hemoglobin digestion and heme dimerization so as to limit accumulation in the DV lumen. Since cell survival depends on rapid crystal growth, nucleation is likely to be enhanced by molecular templates. It was proposed, for example, that biogenic hemozoin grows within a lipid droplet inside the DV,^23,24^ actually mimicking the conditions of *in vitro* growth. It was further proposed that crystals associate with, or even incorporate, lipids and other macromolecules from the surroundings.^25,26^*In situ* studies using soft X-ray cryo-tomography found no evidence of a lipid droplet, however.^3,22,27−29^ Image contrast by this method is specifically sensitive to carbon concentration in the aqueous environment, so it is not possible to hide lipid if present. The spatial resolution is rather modest, however, not better than 25 nm, and this led to a suggestion of a conformal lipid shroud that mediates crystal growth.^24^ Another experimental attempt to find such a shroud was made by cryogenic scanning transmission electron tomography (CSTET).^30−32^ CSTET provides higher spatial resolution, while diffraction contrast is sensitive to crystallinity and reveals the boundary of the hemozoin crystals especially clearly. In these studies, we also noted the typical polar shape of the biogenic crystals, which are less needle-like than the synthetic ones and also show characteristic differences at the two ends. One end is smooth and slanted, like a chisel, while the other is highly variable. Some variable ends are blunt and others jagged; some appear with a sharp overhang, like a pen nib, and others appear distinctly like a handle. Given the translational symmetry of the lattice, this polar shape is not compatible with a centrosymmetric structure and suggests a chiral component in the unit cell.

In this work we enlist the emerging technique of 3D electron diffraction^33−36^ (3D ED) with narrow-field illumination to address the crystalline structure of biogenic hemozoin at higher resolution than had been possible with XRPD. Moreover, the diffraction data are acquired from one or a few crystals at a time, so that the individual lattices can be distinguished. This resolves the ambiguity in powder diffraction analysis between a mixture of isomers within a single phase, on one hand, and a mixture of individually homogeneous crystals on the other. The results indicate that biogenic hemozoin comprises a specific pair of the four possible isomers, with a chiral component that explains the polar morphology. We propose that a bias in the isomer formation optimizes the conditions for rapid crystal growth.

## Results

### In Situ Cryo-Electron Tomography and 3D Electron Diffraction

*Plasmodium*-infected red blood cells were deposited directly on electron microscope grids, blotted to remove excess medium, and then immediately vitrified. Whole-cell cryo-tomography of intact parasites revealed that opposite ends of hemozoin crystals differ: one end shows a smooth chisel shape while the other is highly variable (Figure 1D and Supplementary Movie S1). Large crystals accumulate at the center of the DV, while smaller crystals appear to nucleate around the periphery. This is consistent with prior observations suggesting a site of nucleation at the inner membrane of the DV.^27,37^ A more recent paper correlated the typical shapes with the stages of cell growth: at the earliest stages the crystals grow as thin needles, with the asymmetric trapezoid appearing as the cells mature.^31^ Similar polar shapes have been reported by other authors as well.^38−40^ Observation by cryo-tomography, within the intact DV, eliminates the possibility of mechanical breakage during purification. Therefore, we conclude that this polar form is the native growth morphology for biogenic crystals of hemozoin. A centrosymmetric crystal structure should not produce such a polar morphology.

Two cell preparations were used in the present study. One was grown at 1% ambient oxygen (batch 1) and the other at 5% (batch 2), mimicking conditions for tissue and venous blood, respectively. As discussed below, the aim was to test the possible effect of oxygen retention on the heme prior to dimerization. However, no significant differences were observed between the preparations under low and high oxygen atmospheres, and similar conclusions were reached from both. Details appear in Supplementary Sections 1 and 2.

For diffraction measurements, cells were blotted more aggressively so that the cellular membranes burst, including the DV, and hemozoin crystals spilled onto the surrounding area of the grid. Continuous-rotation 3D electron diffraction data were recorded at −178 °C from isolated crystals that dispersed from the ruptured cells. These included both narrow needles and larger crystals with smaller aspect ratio that come from later growth stages (Table S1). Indexing the crystal faces yielded the surprising result that the end-caps display the (101) facet and a combination of (1̅01̅), (001̅), plus nearby viscinal surfaces on the variable end (Figure 1E, Figure S1). Therefore, we note immediately that the morphology of biogenic crystals differs significantly from that of synthetic β-hematin.

The first complete set of 3992 Friedel-related *hkl* and *h̅k̅l̅* reflections was obtained from 36 individual crystals grown at 1% O_2_. The second data set from 64 crystals grown at 5% O_2_ yielded 8147 unique reflections using an improved detector system (Table S2). In both cases, the scaled intensities of corresponding reflections matched very well despite differences in crystal size and aspect ratio, indicating the presence of only a single crystalline phase for biogenic hemozoin. Moreover, there was a direct correspondence between the number of crystals observed in the search field and the number of lattices observed by diffraction.

**Figure 2:**
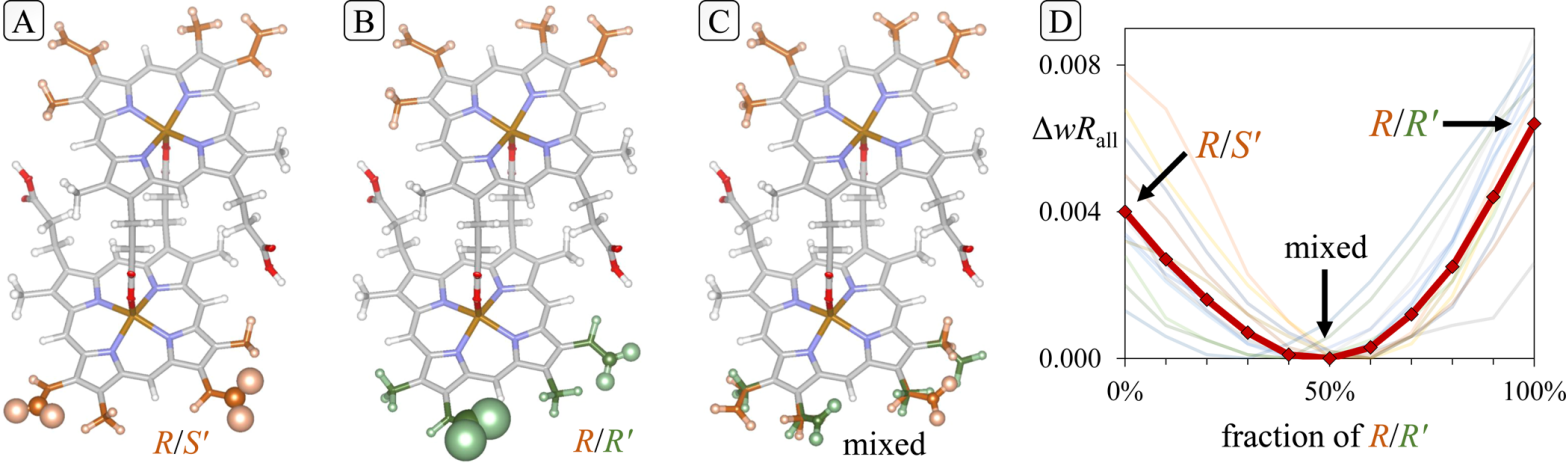
Refinement of the different models reveals a mix of chiral and centrosymmetric dimers. Atom spheres of vinyl sites represent displacement probability surfaces at the 10% level. (A). The *R/S′* model refines well except for the vinyl groups of a single monomer. (B). The chiral *R/R′* model also shows large displacement parameters for the vinyl groups of one monomer. Note the single vinyl group in *trans* conformation. (C). All atoms refine well in a structure containing a mixture of *R/R′* and *R/S′* dimers. Note the *trans* conformation of one *R′* vinyl moiety (in green). (D). *R* factors of dynamical refinements with fixed composition indicate an equal concentration of *R/S*′ and *R/R*′ dimers in the structure. Thirteen faint lines represent the *R* factor for a given composition based on the reflections from individual data sets. The strong line is based on all reflections used in the dynamical refinement with a minimum close to 50% fraction of *R/R′* dimers.

Preliminary analysis and data reduction were performed using DIALS.^41,42^ Lattice parameters of biogenic hemozoin at low temperature were adopted from Straasø et al.^16^ Initial model structures of the three heme dimers, *R/R′*, *R/S′* and *S/R′*, were refined against merged electron diffraction data sets (Supplementary Section 3).^43^ The kinematical refinement cannot distinguish the chiral enantiomers, *R/R′* and *S/S′*, whereas the two centrosymmetric dimers differ in coordination of the propionic acid on the A or D pyrrole to the neighboring Fe (Figure 1). Given the polar crystal habits, the *R/S′* and *S/R′* dimers were not constrained as centrosymmetric entities. Overall *R* factors and relevant atomic displacement parameters (ADPs) appear in Table S3. The fit of the *S/R′* model was considerably worse than the others. The crystal structure model embodying the chiral dimer *R/R′* refined with fewest inconsistencies, while refinement of the *R/S′* model was also satisfactory. In both cases, all parameters refined well except the ADPs of terminal carbons in the vinyl substituents on the B′ and C′ pyrrole groups belonging specifically to the lower (primed) hematin ring (see Figure 2A,B), indicating a loss of atomic localization. By contrast, ADPs of the corresponding carbons on the upper monomer were small and the reconstructed potential appeared sharp.

The discrepancy in ADPs between the two monomers is resolved by a model containing a mixture of dimers, one chiral and one pseudocentrosymmetric (*R/S′*). This accounts for the perfect overlay on one side and the loss of atomic localization on the other. Indeed, for roughly equal dimer occupancy, ADPs for the vinyl substituents were consistent with those of other constituent atoms (see Figure 2C). Notably, too, all the vinyl groups assumed the more prevalent *cis* conformation, except for one on the lower ring that took a *trans* orientation. A further refinement based on dynamical diffraction analysis (Supplementary Section 4) confirmed these conclusions as well as 1:1 occupancy of *R/S′* and chiral dimers, as shown in Figure 2 for the 5% O_2_ data and Figure S5 for the 1% data. The model coordinates of the atoms of the *R/S′* dimer were compared in order to estimate the deviation from a centrosymmetric molecule (Table S5). Terminal carbon atoms of the vinyl groups significantly violate the presence of a center of symmetry, which indicates that the respective local environments of the upper (*R*) and lower (*S′*) monomers must differ significantly.

**Figure 3:**
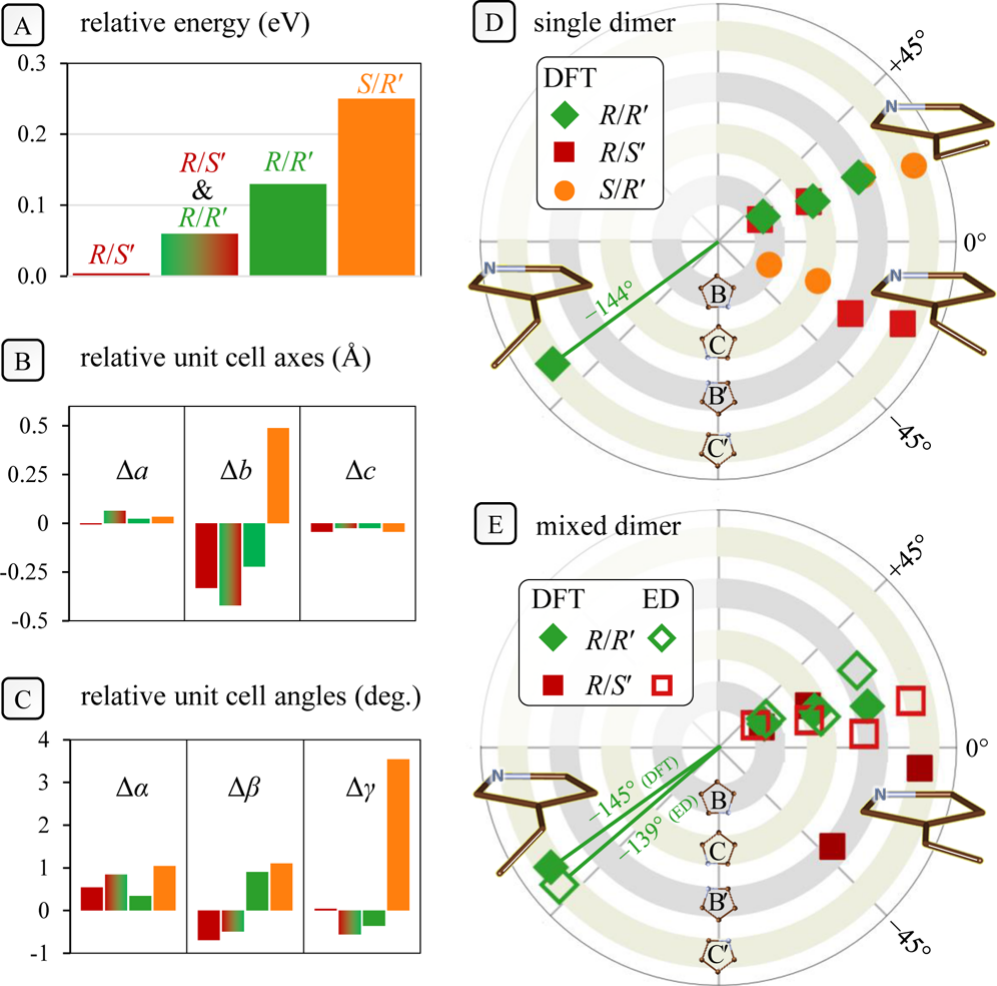
Results of DFT calculations for packing of the various dimers within the unit cell parameters. (A) Energy rankings of the various models tested: *R/S′*, *R/R′*, *S/R′*, and the combined model *R/S′+R/R′*. (B,C) Unit cell parameters of the optimized structures in comparison with XRPD-based parameters.^16^ Color labels as in (A). Note the poor fit of the *S/R′* dimer. (D,E) Polar plots represent dihedral angles for vinyl groups attached to the respective pyrrole rings: B,C for the upper heme, and B′,C′ for the lower heme. A dihedral angle of 0° represents a *cis* orientation of the vinyl perfectly in plane with the pyrrole, whereas 180° signifies a planar *trans* orientation. The key vinyl at the C′ pyrrole is displayed in stick form according to the angle indicated in the polar plots. (D) In a crystal modeled of a single isomer, all vinyl groups take the *cis* conformation except for a single *trans* vinyl on the *R/R′* dimer (see Figure 1 for labels). (E) In the mixed *R/S′+R/R′* crystal model, again DFT (closed symbols) predicts a single *trans* vinyl on the *R/R′* dimer, in agreement with the electron diffraction (ED) experiment (open symbols).

Density functional theory (DFT) calculations with the many-body dispersion (MBD) method^44^ were performed to confirm: a) the plausibility of a crystal containing mixed isomers, and b) the *trans* conformation of one vinyl group. Full unit cell relaxation was performed for crystals composed of pure and mixed dimers. Results are summarized in Figure 3; details appear in Supplementary Section 5. The outcome of DFT+MBD analysis is that the biogenic hemozoin crystal can easily accomodate a mixture of *R/S′* and one chiral dimer, whereas the mixture of two chiral enantiomers or two centro-symmetric dimers is energetically unfavorable. This finding supports the experimentally derived results. Note that the chiral dimers are indistinguishable due to symmetry, so that energy calculations for *R/R′* apply equally to *S/S′.* For the chiral dimer specifically, the DFT calculations indicate that one vinyl on the lower monomer is indeed in a *trans* conformation, while the other three vinyl groups take a *cis* conformation. The dihedral angles of all four vinyl groups at pyrrole sites B, C, B*′*, C*′* are displayed in polar graph form in Figure 3. (For the *S/S′* dimer, the *trans* vinyl would be on the upper, unprimed monomer.) The DFT models for the pure single dimer composition predict a *trans* conformation only for the chiral model, as seen in Figure 3D. The DFT model for the mixed *R/R′* + *R/S′* dimers predicts the single *trans* conformation in agreement with the experimental result, seen in Figure 3E. We attribute this to intermolecular forces in the crystal, which stabilize the otherwise less stable *trans* conformation.

### Polar Morphology and Absolute Structure Determination

**Figure 4:**
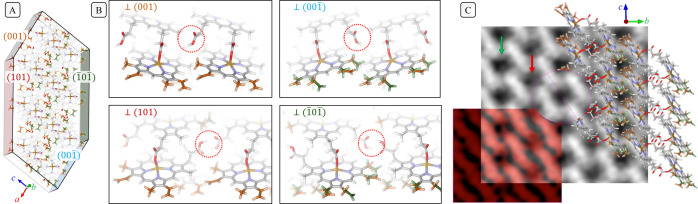
Groups exposed at the surfaces of hemozoin crystals composed of a mixture of *R/S′ + R/R′* dimers. (A). Array of unit cells as a visual guide. The mixture appears as disorder on the (001̅) and (1̅01̅) surfaces, as represented in Figure 2C, but the (001) and (101) surfaces are completely ordered. (B) A detailed view of the methyl-vinyl pairs on the various exposed surfaces. Additionally, the carboxyl and carboxylate groups (dotted red circles) are exposed to the aqueous medium at the (001̅) surface, rendering it more hydrophilic than the (101) and (1̅01̅) surfaces where the available H-bonds are saturated and buried (red circles, fading represents depth). Crystal growth of the relatively hydrophilic (001̅) face will be inhibited by competitive binding of water. (C) Phase reconstruction from a through focus series (gray) of a crystal oriented precisely along the **–*a*** direction. Alternating columns of channels between the heme dimers appear either “filled” (red arrow, carboxyl links) or “empty” (green arrow, methyl-vinyl). The red overlay is a computed phase image based on the diffraction result for the model combining *R/S′ + R/R′* dimers, shown overlaid in ball and stick form. The faint purple circle shows one methyl-vinyl channel; note the asymmetric density around the dashed line, whose origin lies in the chiral contribution. After indexing as indicated, the chiral dimer is identified as *R/R′*. For more details, see Supplementary Section 6.

We may deduce the absolute structure on the basis of morphology and the intrinsic atomic disorder in specific orientations.^45^ A crystal composed purely of centrosymmetric *R/S′* dimers should adopt a nonpolar habit with symmetric ends. As seen in Figure 1, the habit is polar, with a (101) face at the +***c*** end and often a short (001̅) face at the **–*****c*** end; other crystals display a mixed shape at –***c*** with a ragged (001̅) facet and a decidedly longer handle-shaped segment capped by a (1̅01̅) facet (Figures 1D and S1). The (101) and (1̅01̅) surfaces run along a plane of the dimers in the ***a–c*** orientation. Considering that the carboxyl pairs form H-bonded acid chains in precisely this direction, water in the aqueous medium of the digestive vacuole will bind poorly to either face. By comparison, the carboxyl and carboxylate groups at the (001̅) face are well exposed (Figures 4 and S6–S8), so that growth of this face will be inhibited by competitive binding of water molecules.^46^ Growth of the (001) face could similarly be inhibited relative to the (101). Note, however, that the (001̅) and (001) faces differ essentially in terms of the exposed disorder. Considering the mixed crystal to be composed of *R/S′ + R/R′* dimers, atomic disorder will occur at the **+*****b*** face and the **–*****c*** end of the rod, where the shape variability in fact appears. (By contrast, the *R/S′ + S/S′* mixture would be disordered on the **–*****b*** face and the **+*****c*** end.) Local inhomogeneities could then explain the variable formation of the (1̅01̅) and (001̅) facets, where the latter grows more slowly.

We next attempted to confirm the morphological analysis experimentally. Kinematical diffraction analysis cannot distinguish chiral handedness, i.e., *R/R′* or *S/S′*. In principle, the interference of simultaneously excited beams within the crystal makes the diffracted intensities sensitive to the absolute structure.^47^ In the present case that would enable identification (*R/R′* or *S/S′*) of the chiral dimer. The effect is weak, however, given that mainly the terminal, half occupied vinyl CH_2_ groups contribute to the chirality, and we have been unable to discriminate the handedness with presently available methods of dynamical diffraction analysis (Table S6).

In an alternative approach, we addressed the issue of absolute structure by a newly developed transmission EM method based on through-focus imaging and phase retrieval.^48^ Along the **–*****a*** direction, the vinyl-methyl groups are oriented as atomic columns within otherwise empty channels. Crystals were imaged at sufficient resolution to observe the lattice fringes, and computed Fourier Transforms were examined in order to find one suitable for the full analysis (Supplementary Section 6). After phase reconstruction, an asymmetry in the channel could be seen clearly, with one blunt protrusion and a second one that was sharper but off-center. This asymmetry is expected for a chiral dimer. Identifying the handedness still required a determination of the crystal axes, however, as the real space analysis cannot distinguish the ***+a*** from ***–a*** directions. Therefore, the identical crystal was located again on the grid and a diffraction tilt series was recorded. In order to avoid potential ambiguities in software, the crystal was indexed using the same DIALS pipeline as above. Simulated images were then generated by multislice methods using the refined models generated in the dynamical diffraction analysis, and a similar asymmetry in the channels was observed. Figure 4 summarizes these results with an overlay of the ball-and-stick model over the simulation, over the reconstructed phase image. The orientation of the asymmetry with respect to that of the crystal lattice indicated a mixture containing the *R/R′* rather than the *S/S′* chiral dimer, confirming the morphological analysis presented above.

### Macromolecular Inclusions and Lattice Distortions

It has been proposed that biogenic hemozoin should incorporate a considerable fraction of organic macromolecules, such as neutral lipids and cholesterol, or even proteins, from the complex growth environment.^25^ Such incorporation would have to be reflected in distortions or defects in the crystal lattice, which is apparently at odds with the high resolution obtained in the crystallographic analysis (better than 1 Å for both high and low oxygen data sets; Supplementary Section 3). Nonetheless, we invoked a number of tests to look for lattice distortions. The simplest was a measure of possible microdomain misorientation, or mosaicity, determined automatically as part of the diffraction data reduction. The median value of 0.08° (Supplementary Table S4) is small in comparison with other organic and inorganic crystals analyzed similarly by 3D ED.^47^ The second involved analysis of the high-resolution TEM images recorded for the purpose of absolute structure determination described above (Supplementary Section 6). A Fourier Transform was computed on a sliding window over the lattice images of several crystals, including the one used for handedness determination. Lattice strains would appear sensitively as geometrical distortions of the patterns, but only the relative intensities of the spots change as the window is scanned across the larger image (Supplementary Movies S3,S4). The visual test was complemented with a quantitative geometrical phase analysis, which detected a variability in lattice spacing on the order of ±1% throughout the crystal (Supplementary Figure S11). Finally, hemozoin crystals were analyzed by 4D STEM measurements (Supplementary Figure S12). Diffraction projections were recorded as a local probe scanned across the crystals. The simultaneously recorded high-angle scattering reveals a homogeneous mass density and composition. A tilt of the single crystal’s zone axis on the order of 1° - 2° is associated with a subtle bending of the rod-like crystals and a minor homogeneous strain. Altogether these results are consistent with expectations for a pure crystal. No evidence is detected for dislocations, voids, or other defects that would suggest the presence of engulfed foreign macromolecules.

## Discussion

The polar morphology of biogenic hemozoin crystals provides a strong first indication for a chiral component in the unit cell. A particularly esthetic example appears in Figure 1 and Supplementary Movie S1. Perhaps the most notable comparison with β-hematin is that the biogenic crystals are more regular in shape and size than the synthetic ones. Moreover, the growth morphology is different. We can attribute the different facets to solvent-crystal surface interactions. In organic solvent, β-hematin grow primarily as long, thin laths with atomically smooth surfaces. The broad {100} faces are capped by {011} faces at the ends. This is consistent with a theoretical prediction based on orientation-dependent attachment energies.^14^ The biogenic crystals, on the other hand, are subject to interaction with the surrounding water at the exposed carboxyl and carboxylate moieties. Synthetic crystals are capped by (011) and (01̅1̅) facets, which did not appear at all in the biogenic hemozoin. We note that these surfaces can bind water (see Figure S8) so their growth should be inhibited although they are atomically smoother and energetically preferred. We observe instead the (101) and (1̅01̅) planes capping the crystal ends. These facets cannot bind water and are therefore favored for hemozoin. This sensitivity of the morphology to growth in water is further evidence for the lack of an engulfing lipid medium.

The most striking observation in the crystal structure analysis is the asymmetry between the two monomers in the unit cell, which reflects the mixture of two of the four possible isomers that can form by dimerization. The identity of the chiral dimer, i.e., the absolute structure *R/S′ + R/R′*, was determined by morphological analysis based on inhibition of growth at the (001̅) face, and confirmed by analysis of focal series phase reconstruction. The latter recalls assignment of molecular chirality in centrosymmetric crystals composed of a mixture of chiral *R* and *S* molecules grown in the presence of a tailor-made chiral inhibitor (*R′* or *S′*).^45^ In this regard, we present an exceptional assignment of handedness for a molecule of unknown chirality.

Given the impossibility to accumulate a substantial concentration of heme in water, hemozoin crystallization must occur as rapidly as the hemoglobin digestion and heme dimerization steps. This corresponds to an “assembly line” model of crystallization^22^ whereby the crystal growth is not rate limiting, in contrast to a quench from supersaturated solution. DFT calculations predict a similar formation energy for the *R/S′* and chiral isomers in isolation, with only the *S/R′* dimer disfavored.^51^ (Indeed, the pro-chiral vinyl moieties on opposite sides of the hematin dimer do not sterically interact.) While thermodynamically all four dimers may form, we find only two in the hemozoin crystals. This observed selection of the dimer requires involvement of a chiral agent, or catalyst, in the dimerization mechanism, i.e., prior to incorporation into the crystal. An earlier model proposed that the selection is imposed by persistent oxygenation of the central iron ion.^16,22^ Heme iron is oxygenated on the *R* face in hemoglobin. If oxygen remains bound after degradation of the protein, it would inhibit dimerization specifically on that side. As a test, the diffraction studies were conducted on crystals from cells that had been grown under two different oxygen environments. According to the oxygen-binding properties of hemoglobin, 1% ambient O_2_ corresponds to heme oxygenation below 10%, whereas 5% O_2_ provides a saturation of 75%. In both cases, however, the diffraction analysis indicated a similar concentration of about 50% chiral dimer. Had persistent oxygenation played a role in chiral isomer selection, we would have expected to see a significant difference in the fraction of the chiral dimer. Absent such an observation, the present data do not support this hypothesis.

**Figure 5:**
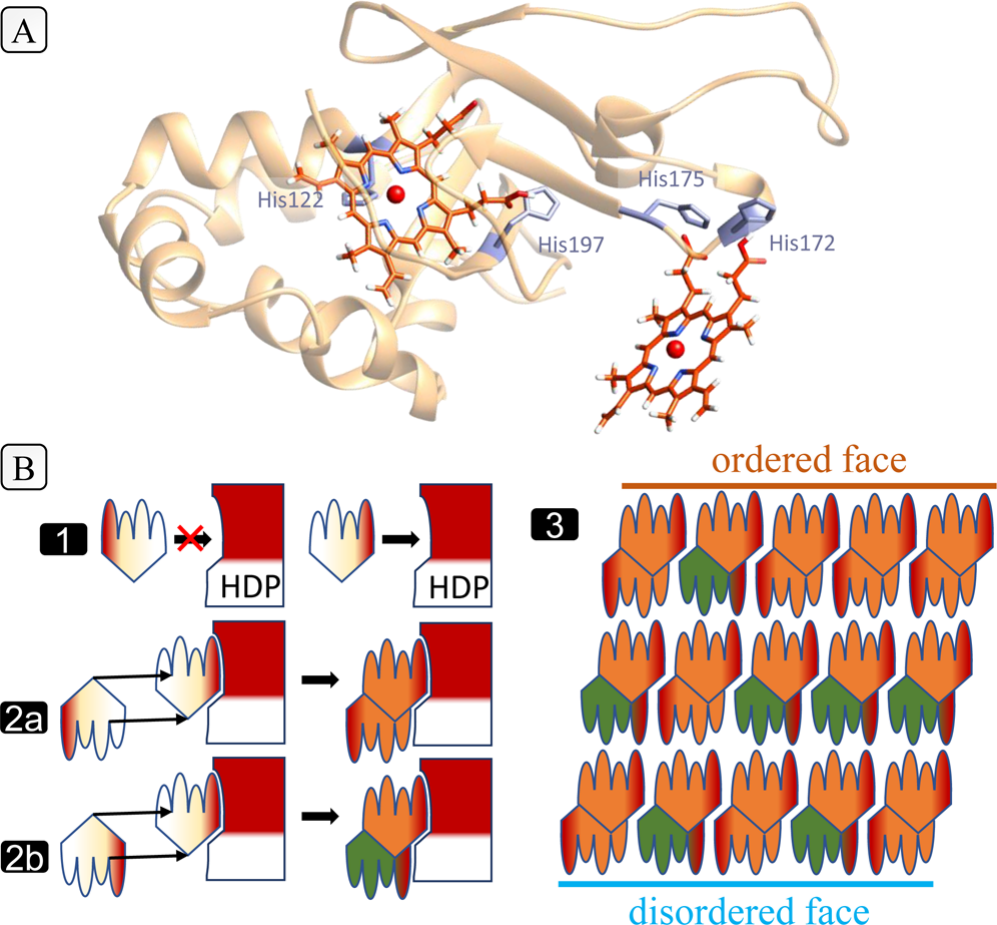
Model for selection of the chiral dimer and its relation to surface disorder. (A) A predicted structure for the heme detoxification protein (HDP) generated by AlphaFold^49^ with heme groups docked manually to reflect known histidine interactions.^50^ (B) A topological model for the function of HDP. A chiral agent that preferentially orients one monomer generates one centrosymmetric (step 2a) and one chiral dimer (step 2b). A crystal of these dimers (step 3) is necessarily polar, with atomic disorder appearing on only one side.

Another hypothesis centers on a protein-based mechanism to promote selective dimerization. The heme detoxification protein (HDP) is associated with falcipain 2 and other proteases. It has been implicated in formation of cyclic dimers^52−54^ and was shown to accelerate the transformation of heme to hemozoin *in vitro*. Therefore, we checked whether it offers a plausible selection mechanism. Biochemical studies have identified four key histidine residues: His122 and His197 form one heme binding site, with His122 as the ligand to iron, while His172 and His175 form another.^50,55^ To date there is no high-resolution molecular structure available for HDP, although it is recognized to have a fascilin-like fold. Therefore, we turned to structural modeling by AlphaFold^49^ and other servers (Figure 5A and Supplementary Section 7). There was a broad consensus at high confidence in the architectural features, with a compact α-helical domain at the N terminus and a largely unfolded cap at the C terminus. One heme binding site containing the Fe-binding histidine appears in a pocket between the compact core of the protein and the cap, while the other appears at the tip of a long unfolded loop. Specifically, the site spanning His122 and His197 appears in the pocket between the core and the cap. Presuming that the heme Fe binds to His122, as found earlier by spectroscopy,^50^ and one of the propionic acid tails binds to His197, the vinyl/methyl pairs are oriented deeply in the protein structure where chiral discrimination is likely to take place. The other heme binding site consisting of His172 and His175 appears at the tip of an unfolded loop. These histidines were not found to bind the Fe. If chiral discrimination at the protein core fixes the orientation of one heme, then only one chiral dimer may form (Figure 5B, Figure S14).

An independent study used the I-TASSER server to generate a structural model for HDP, which was similar overall to the AlphaFold prediction. The authors identified putative heme binding regions on the basis of short sequence homology with other histidine-rich proteins.^56^ These regions largely coincided with the previously identified ones, although not with His122 residue that was isolated in sequence but proximal in both three-dimensional models. The authors proposed a different binding configuration, but they too suggested one site buried in a protein pocket and the second flexibly bound at the end of the same unfolded loop. The latter site may be involved in a hand-off mechanism for dimerization, dependent on large-scale movements, or it may serve simply to enhance the average heme concentration locally.

Details of the model depend on structure prediction for the implicated heme detoxification protein, but the proposition is topologically robust: chiral recognition in the binding of one monomer is sufficient to enhance formation of one chiral dimer over the other. Given the uncertainty in binding orientation, it would not be possible to predict which chiral dimer should be preferred. In principle, both centrosymmetric isomers could be generated as well (Figure S13). Note, however, that the diffraction data analysis as well as the DFT results suggest that the *S/R′* dimer would crystallize with unit cell parameters different to those observed experimentally. It is likely that additional steric constraints distinguish between the centrosymmetric dimers.

According to DFT calculations, the mixture containing the *R/S′* plus one chiral dimer should cocrystallize with minimal energetic penalty. Other hypothetical combinations entail a significantly higher energy than the pure *R/S′* model (Supplementary Figure S9). Thus, the significance of the dimerization catalyst is apparently to accelerate hemozoin formation by preselection of the isomers that will mix well in the crystal lattice. Incorporation of the other isomers would necessarily induce a lattice strain and slow the crystal growth. This effect might also explain the greater variability in crystal shapes and sizes for synthetically grown β-hematin, lacking such a selection mechanism.

In light of the present results we can reconsider the implications of biogenic hemozoin growth within a complex macromolecular environment.^24,26,38^ Surface-specific analysis reveals a rich spectrum of macromolecular adsorption,^57^ and it was suggested that hemozoin associates with or even engulfs certain lipids, cholesterol, and proteins within the crystalline lattice.^23,25^ Supporting evidence comes from biochemical separations of the crystals in bulk, followed by sensitive detection analytics such as mass spectrometry. While it is clear from the literature on biomineralization that single crystals may incorporate foreign components, this cannot occur without effect on the lattice.^58^ Indeed, crystallization is a standard method for purification in chemical synthesis, notably in the pharmaceutical industry. Therefore, we dedicated significant effort to resolve the nature of biogenic hemozoin, either as an essentially pure crystal or as a relatively open lattice that accommodates other organic macromolecules.

Impurities within the crystal volume may concentrate in voids, at grain boundaries, or at vacancies or interstitial sites in the lattice. Given that the putative inclusions are comparable in size to the unit cell, the hydrogen-bonded chains linking them along the ***a-c*** direction would have to be disrupted. As such, it is very difficult to reconcile a conceptual picture of hemozoin as a spongy structure capable of engulfing lipids or proteins with the high-order Bragg reflections observed. The overall resolution, better than 1 Å, is superior to all diffraction experiments on β-hematin reported to date.^16,21,59^ The median mosaicity of 0.08° of the specimen hemozoin crystals is also small in comparison with typical mosaicity values of inorganic and organic compounds measured by 3D ED.^47^ Notably, only 4 tilt series of 51 in the two batches were excluded for technical reasons from the analysis; the sharp patterns are clearly the typical ones. Macromolecular inclusions would also cause lattice distortions to appear in high resolution TEM imaging. Both visual and quantitative analysis revealed only a very small variability in lattice parameters, inconsistent with significant distortion that would be induced by molecular inclusions. Scanned diffraction with a local probe, i.e., 4D STEM, also revealed only a subtle tilt of the zone axis. Results of these tests, individually and collectively, are not consistent with accumulation of engulfed material that would have to disrupt the hydrogen bonding along the ***a-c*** direction, or otherwise accumulate in voids.

Crystallization of hemozoin is apparently a flexible mechanism for heme detoxification. It is common to all species of *Plasmodium*, though perhaps with subtle differences between them. For example, knockout of a lipocalin-family protein leads to strongly branched growth of hemozoin in *P. falciparum*, but instead to twinned growth in *P. berghei*.^22,60^ Hemozoin forms as well in a number of other blood-feeding organisms, including the blood fluke *Schistosoma mansoni* and the kissing bug *Rhodnius prolixus*. In contrast to *Plasmodium*, in these cases the crystal growth environment is extracellular.^61^ Even in mice, disruption of the heme oxidation pathway can lead to deposits of hemozoin in macrophage cells.^62^ It will be interesting in future to explore and compare the structure of the crystals formed in these various biogenic contexts.

In summary, cryo-tomography, 3D electron diffraction, density functional theory, morphological analysis, through-focus imaging with phase reconstruction, and nanoprobe diffraction combine to yield a refined structure of native hemozoin from *Plasmodium falciparum*. The approach offers a combination of experimental and analytical methods at the state of the art in electron microscopy. We conclude that the biogenic crystals contain a 1:1 mixture of dimers, one centrosymmetric and one chiral, with atomic disorder in the **–*****c*** and **+*****b*** orientations. The facets include previously unrecognized, deeply corrugated (101) and (1̅01̅) surfaces. The growth morphology reflects the solvent inhibition by competitive binding of water to exposed carboxyl groups. Inhibition of hemozoin nucleation and growth is a major target of antimalarial drugs, both via formation of soluble drug-heme adducts^4^ and by direct surface adsorption to poison growth of the crystal.^22,63^ To date the synthetic β-hematin has served as a model for drug interaction studies. Clarification of the facets and structure of the biogenic form, at much improved level of detail, should serve as a template for development of drug treatments for malaria where hemozoin is a target.

## Data Availability

All data needed to evaluate the conclusions in the paper are present in the main text and/or the Supporting Information. Diffraction data sets are published at 10.5281/zenodo.5039355 and 10.5281/zenodo.7462145. Crystal structure models have been deposited at the Cambridge Structural Database under deposition numbers 2240526, 2156990, and 2344281. The cryo-STEM tomographic reconstruction shown in Figure 1 is available at EMDB under ID: EMD-50857.

## Supporting Information Available

Extensive descriptions of the experimental and analytical methods appear in the Supporting Information under the following major headings: Experimental Methods, Crystal Morphology and Indexing, Structure analysis and kinematical refinement, Dynamical Diffraction Analysis, Density Functional Theory, High-resolution TEM, Focal Series Reconstruction, and Nanoprobe Diffraction, Model for oriented dimer formation by Heme Detoxification Protein, and Captions for Supplementary Movies S1–S4 (PDF)
Movie S1 (AVI)
Movie S2 (MP4)
Movie S3 (MP4)
Movie S4 (MP4)
Transparent Peer Review report available (PDF)

## Supplementary Material

oc4c00162_si_001.pdf

oc4c00162_si_002.avi
