# Supplementary material for: Cryo-tomography and 3D Electron Diffraction Reveal the Polar Habit and Chiral Structure of the Malaria Pigment Crystal Hemozoin
Source: ACS Cent Sci. 2024 Jul 4;10(8):1504–14. doi: 10.1021/acscentsci.4c00162 (PMC11363319; doi:10.1021/acscentsci.4c00162)
Supplement: Supplementary file 6 — oc4c00162_si_006.pdf [file oc4c00162_si_006.pdf]

oc-2024-00162y.R1

Name: Peer Review Information for "Cryo-tomography and 3D Electron Diffraction Reveal the Polar Habit and Chiral Structure of the Malaria Pigment Crystal Hemozoin"

First Round of Reviewer Comments

Reviewer: 1

Comments to the Author

Klar et al reported the characterization of the chiral structure of biogenic hemozoin crystals using cryo-tomograph and 3D electron diffraction approaches. The reviewer is not an expert in the latter so the editor/authors should refer to other reviewers for relevant comments. Rather, the following comments are from the perspective of an interested structural biology colleague. The current manuscript is not well-written to explain the authors' discoveries to the general audience.

By reading the Abstract, it is unclear what is the most supported discovery (not even mentioning the R/R' found later in the results). Throughout the manuscript, the characterizations were mixed with hypothesis and results so it is unclear to judge what is the most confident conclusion based on the data, what is just "consistent" with one model over the others.

Please comment on the switching of polarity for the crystals in the tomograms (Movie S1: 00:26 s vs 00:38 s, the former one is the similar to Fig. 1D while the latter seems to have the polarity flipped for the three crystals). It seems that the crystals at different z are connected, but they just happen to switch polarity at the same plane?

Fig. 3 AB are not well labeled so it's unclear what do the axes mean.

It is unclear what does the Fig. 4G mean with the different colors and shapes without labels.

If the biogenic crystals are a mixture of stereoisomers, please comment how could this information serve as a guide to new drug development as mentioned in the last sentence of the Abstract (at least in the discussion).

Reviewer: 2

Comments to the Author

The article by Klar and Co. is a powerful demonstration of the application of new electron microscopy and electron diffraction methods. It presents a detailed and methodical dissection of the atomic structures within the malarial pigment crystals. Overall, the article is suitable for publication pending minor revisions, as follows:

The authors apply phase retrieval and multi-slice methods to determine the absolute structure of the hemozoin crystals, but note that, in addition to the few atoms dictating the structural difference between enantiomorphs, "The simplistic modelling of the disorder leads to further deviations of the calculated intensities and worsens the fit. Furthermore, inelastic scattering is not taken into account and it is assumed that the crystal is perfect. Crystal defects and inelastic scattering are thus not only responsible for the relatively high R-factor, but in this case hinder the determination of the absolute structure."

While the logic for these reasons seems sound, the evidence in the case of these crystals is slim. The degree of inelastic scattering isn't well characterized, nor is the specific thickness of the crystals measured (and its correlation to the correctness of absolute structure prediction). To what degree is radiation damage at play for these crystals, as well?

The authors also note that the crystals diffracted to 1Å and implied that the resolution was indicative of highly ordered, crystals. However, from their own Figure S1, one can detect obvious and beautiful bend contours within the crystals that no doubt indicate imperfections in the lattice. The authors' point would therefore be much more convincing with diffraction-based evidence of lattice regularity, e.g. as indicated by scanning diffraction methods such as 4DSTEM.

That approach would significantly bolster the claims of variability in lattice spacing deduced from visual inspection of high resolution images. In addition, it would better indicate out of plane lattice distortions than the imaging would.

Reviewer: 3

Comments to the Author

The authors of “Cryo-tomography and 3D Electron Diffraction Reveal the Polar Habit and Chiral Structure of the Malaria Pigment Crystal Hemozoin” showcase a fantastic collection of results obtained from cutting edge techniques. The detailed discussion of 3D ED and the associated modeling approaches was extremely thorough; the HRTEM imaging of crystals enabled real-space studies of the hemozoin structure, as well as evidence suggesting the 1:1 mixture of dimers, one centrosymmetric and one chiral. These results are bolstered by the DFT analysis and confirmed the plausibility of the mixture. I have very few critical comments on this work, and I believe this report will quickly become popular to those interested in structural science and malaria treatment.

The conclusions are somewhat undersold, and the dynamical diffraction study that corroborates the tomography work should be mentioned. The supporting information contains a wealth of knowledge. However, I think more information could be shared. There are CCDC deposition numbers for the dynamical diffraction models, but have the authors shared the uncorrected, kinematical HKL files and associated models? Many of those refinement metrics are presented (Table S3), it would be nice to correlate them to the full structure determination.

Author's Response to Peer Review Comments:

Dear Editors,

Please find the revision of our manuscript “Cryo-tomography and 3D Electron Diffraction Reveal the Polar Habit and Chiral Structure of the Malaria Pigment Crystal Hemozoin”. We thank all the referees for their careful reading and thoughtful comments. In the following we address the changes and replies with text highlighted in yellow. A “Tracked changes” version of the manuscript and supplementary information files were uploaded as Supplementary information.

Sincerely,
Michael Elbaum

Formatting Needs:

MS Files: Complete author names are not provided. (Klar, Paul Benjamin, Waterman, David Geoffrey)
Middle names have been added for Klar, Waterman, and Gilchrist. David Owen prefers the current listing.

MS Files: Abstract contains more than 200 words.
The abstract was shortened.

MS Files: Associated Content/Supporting Information paragraph is missing.
I did not find this described in the author guidelines. A list of the Supplementary Information contents appears after the References in the main text. Is this sufficient?

MS Files: Synopsis is missing.
A synopsis was added below the graphical abstract.

SI Files: Page numbered as 1,2, 3, ... instead of S1, S2, S3, ...
Fixed.

Reviewer(s)' Comments to Author:

Reviewer: 1

Recommendation: Does not meet the requirements of publishing in ACS Central Science.

Comments:
Klar et al reported the characterization of the chiral structure of biogenic hemozoin crystals using cryo-tomograph and 3D electron diffraction approaches. The reviewer is not an expert in the latter so the editor/authors should refer to other reviewers for relevant comments. Rather, the following comments are from the perspective of an interested structural biology colleague. The current manuscript is not well-written to explain the authors’ discoveries to the general audience.

By reading the Abstract, it is unclear what is the most supported discovery (not even mentioning the R/R’ found later in the results). Throughout the manuscript, the characterizations were mixed with

hypothesis and results so it is unclear to judge what is the most confident conclusion based on the data, what is just “consistent” with one model over the others.

The abstract had to be shortened due to the 200 word length limitation, but this feedback motivated a reordering of several paragraphs to improve the distinction between results and discussion.

Please comment on the switching of polarity for the crystals in the tomograms (Movie S1: 00:26 s vs 00:38 s, the former one is the similar to Fig. 1D while the latter seems to have the polarity flipped for the three crystals). It seems that the crystals at different z are connected, but they just happen to switch polarity at the same plane?

There are actually two parallel layers of separate crystals. What appears as flipped polarity is simply another crystal in the opposite orientation. An explanation was added to the video caption in order to clarify this point.

Fig. 3 AB are not well labeled so it’s unclear what do the axes mean.

The caption was rewritten to clarify that the polar plot represents the dihedral angles for vinyl groups.

It is unclear what does the Fig. 4G mean with the different colors and shapes without labels.

We have adapted the numbering of the steps, changed slightly the colors and added a clarification in the caption for Fig. 4G.

If the biogenic crystals are a mixture of stereoisomers, please comment how could this information serve as a guide to new drug development as mentioned in the last sentence of the Abstract (at least in the discussion).

The design of surface-binding crystal growth inhibitors depends on the orientation and nature of the exposed facets. A comment to this effect was added to the summary paragraph. In this sense, the expectations drawn from synthetic β-hematin may be misleading.

Additional Questions:
Quality of experimental data, technical rigor: High

Significance to chemistry researchers in this and related fields: Top 5%

Broad interest to other researchers: High

Novelty: High

Is this research study suitable for media coverage or a First Reactions (a News & Views piece in the journal)?: Yes

Reviewer: 2

Recommendation: Publish in ACS Central Science after minor revisions noted.

Comments:
The article by Klar and Co. is a powerful demonstration of the application of new electron microscopy and electron diffraction methods. It presents a detailed and methodical dissection of the atomic structures within the malarial pigment crystals. Overall, the article is suitable for publication pending minor revisions, as follows:

The authors apply phase retrieval and multi-slice methods to determine the absolute structure of the hemozoin crystals, but note that, in addition to the few atoms dictating the structural difference between enantiomorphs, "The simplistic modelling of the disorder leads to further deviations of the calculated intensities and worsens the fit. Furthermore, inelastic scattering is not taken into account and it is assumed that the crystal is perfect. Crystal defects and inelastic scattering are thus not only responsible for the relatively high R-factor, but in this case hinder the determination of the absolute structure."
While the logic for these reasons seems sound, the evidence in the case of these crystals is slim. The degree of inelastic scattering isn't well characterized, nor is the specific thickness of the crystals measured (and its correlation to the correctness of absolute structure prediction). To what degree is radiation damage at play for these crystals, as well?

Our original presentation of the absolute structure determination was perhaps misleading in pointing out first the failure of dynamical diffraction analysis to provide an unambiguous identification of the chiral dimer. In the revision, we open the topic with the morphological argument that is independent of experimental details (other than indexing). We next describe briefly the unsuccessful attempt to confirm the conclusion by dynamical diffraction analysis, with a more complete description in the supplementary information. Our conclusion from the morphology was successfully confirmed by a real-space analysis based on through-focus imaging and phase retrieval, which is independent of the shortcomings that precluded determination by dynamical analysis.

We also appreciate that it was not clear if the quoted limitations refer to the dynamical refinement, to the sample, or to our assumptions. We therefore rewrote the relevant paragraph on p S19 in the SI.

The authors also note that the crystals diffracted to 1Å and implied that the resolution was indicative of highly ordered, crystals. However, from their own Figure S1, one can detect obvious and beautiful bend contours within the crystals that no doubt indicate imperfections in the lattice. The authors' point would therefore be much more convincing with diffraction-based evidence of lattice regularity, e.g. as indicated by scanning diffraction methods such as 4DSTEM.
That approach would significantly bolster the claims of variability in lattice spacing deduced from visual inspection of high resolution images. In addition, it would better indicate out of plane lattice distortions than the imaging would.

We are well equipped today for 4D STEM measurements, so we have added a nanoprobe diffraction measurement to the supplementary information section dealing with lattice regularity. The results show that the bend contours result from a subtle tilt of the lattice rather than loss of regularity. The bend contour contrast was accentuated by selecting diffraction from the 100 orientation, and then we examined regions of maximal and minimal intensity. A diffraction pattern simulated from the coordinates determined by the 3D ED refinement fit perfectly in both areas, simply with a slight tilt of the view angle. As the reviewer suggested, in this particular crystal the strain is in the out of plane

direction and would be hard to detect visually. Still, we conclude that the crystal lattice is essentially perfect with a minor strain that leaves no room for macromolecular incorporation.

Additional Questions:
Quality of experimental data, technical rigor: Top 5%

Significance to chemistry researchers in this and related fields: Top 5%

Broad interest to other researchers: Top 5%

Novelty: Top 5%

Is this research study suitable for media coverage or a First Reactions (a News & Views piece in the journal)?: Yes

Reviewer: 3

Recommendation: Publish in ACS Central Science after minor revisions noted.

Comments:
The authors of "Cryo-tomography and 3D Electron Diffraction Reveal the Polar Habit and Chiral Structure of the Malaria Pigment Crystal Hemozoin" showcase a fantastic collection of results obtained from cutting edge techniques. The detailed discussion of 3D ED and the associated modeling approaches was extremely thorough; the HRTEM imaging of crystals enabled real-space studies of the hemozoin structure, as well as evidence suggesting the 1:1 mixture of dimers, one centrosymmetric and one chiral. These results are bolstered by the DFT analysis and confirmed the plausibility of the mixture. I have very few critical comments on this work, and I believe this report will quickly become popular to those interested in structural science and malaria treatment.

The conclusions are somewhat undersold, and the dynamical diffraction study that corroborates the tomography work should be mentioned. The supporting information contains a wealth of knowledge. However, I think more information could be shared. There are CCDC deposition numbers for the dynamical diffraction models, but have the authors shared the uncorrected, kinematical HKL files and associated models? Many of those refinement metrics are presented (Table S3), it would be nice to correlate them to the full structure determination.

We thank the reviewer for the positive comment. We do believe that the work is an important achievement, which we tried to summarize in the last paragraph without overselling: "In summary, cryo-tomography, 3D electron diffraction, density functional theory, morphological analysis, through-focus imaging with phase reconstruction, and nanoprobe diffraction combine to yield a refined structure of native hemozoin from *Plasmodium falciparum*." Indeed, the dynamical refinement is not emphasized in the manuscript, but it is clearly indicated where it was relevant in the text and especially in the figure captions (Figs. 2 and 3).

As suggested by the reviewer, we submitted the most relevant model from the kinematical refinement (R/S' + R/R') to the CSD (deposition number 2344281) and provide the CIF files of the

models mentioned in the main article (3 CIF files from batch 1, 3 CIF files from batch 2) as supplementary files. We believe that this selection provides all relevant results from the data analysis within the kinematical theory of diffraction. We are happy to share all models mentioned in the SI (about 360 MB) upon request. We also note that the raw data are publicly available on Zenodo and the links are provided in the SI.

Additional Questions:
Quality of experimental data, technical rigor: Top 1%

Significance to chemistry researchers in this and related fields: Top 1%

Broad interest to other researchers: Top 5%

Novelty: Top 1%

Is this research study suitable for media coverage or a First Reactions (a News & Views piece in the journal)?: Yes

oc-2024-00162y.R2

Name: Peer Review Information for "Cryo-tomography and 3D Electron Diffraction Reveal the Polar Habit and Chiral Structure of the Malaria Pigment Crystal Hemozoin"

Second Round of Reviewer Comments

Reviewer: 1

Comments to the Author

Klar et al. described the chiral structure of biogenic hemozoin crystals using cryo-tomography and 3D electron diffraction techniques. However, the discovery/results and discussion were not clearly distinguished so the manuscript is very hard to understand, making it hard to judge the quality of science.

For example:

In the Abstract, “serve as a guide to new drug development” is very vague and seems to be an overstatement. Afterall, there are only few sentences about this in the discussion.

The introduction lacks proper descriptions of biology – how’s hemozoin formed and what are the roles of key residues. Mentioning some of the information in the results section is abrupt and not helpful for the general audience.

The entire “Chiral dimer formation” is filled with ideas from the authors or referring to previous studies, but rarely any actual results. Most of it should be in discussion.

Fig. 3 AB are still not well explained for the general audience (legends).

“Results of DFT calculations for packing of the various dimers within the unit cell parameters. Polar plots represent dihedral angles for vinyl groups attached to the respective pyrrole rings (??? What are the axes?): B,C for the upper heme, and B',C' for the lower heme.”

Reviewer: 3

Comments to the Author

I thank the authors for replying to my comments and depositing the kinematical refinement. I believe the manuscript is ready for publication without changes.

Author's Response to Peer Review Comments:

Dear Editors, please find below, in green font, our replies to the comments of Reviewer 1. The expanded introduction should make the message more accessible to a broad audience. We believe that the formatting needs had been addressed in the first revision. A version of the word document with “tracked changes” enabled is attached as supplementary material for review. We hope that the manuscript is now ready for publication.
Sincerely,
Michael Elbaum

Reviewer(s)' Comments to Author:

Reviewer: 1

Recommendation: Does not meet the requirements of publishing in ACS Central Science.

Comments:

Klar et al. described the chiral structure of biogenic hemozoin crystals using cryo-tomography and 3D electron diffraction techniques. However, the discovery/results and discussion were not clearly distinguished so the manuscript is very hard to understand, making it hard to judge the quality of science.

The manuscript draft was written initially with combined results and discussion and later edited to the separate section form. The separation has been strengthened in the present version.

For example:

In the Abstract, “serve as a guide to new drug development” is very vague and seems to be an overstatement. Afterall, there are only few sentences about this in the discussion.

Drugs inhibiting crystal growth should bind to the crystal surface. To date, the synthetic beta-hematin has been used as a convenient experimental model for hemozoin. Beta-hematin is grown *in vitro* in a low polarity solvent, however, and we report that the exposed facets are significantly

different in the biogenic crystal. The {011} orientations, in particular, do not appear at all. These have been regarded as the fast growing directions because of the needle-like form, but the aspect ratio of the biogenic crystals

actually decreases as they grow larger. The isomeric mixture implies that the $(\bar{0}01)$ and (010) facets display atomic scale disorder. Furthermore, the sensitivity of the morphology to the growth environment provides additional evidence for growth in water rather than a surrounding lipid medium. This, too, is relevant for drug design.

The introduction lacks proper descriptions of biology – how's hemozoin formed and what are the roles of key residues. Mentioning some of the information in the results section is abrupt and not helpful for the general audience.

The introduction has been expanded significantly to explain the biological context, as well as the relevance to drug action.

The entire "Chiral dimer formation" is filled with ideas from the authors or referring to previous studies, but rarely any actual results. Most of it should be in discussion.

This section was moved to the discussion as requested. Accordingly, the relevant panels were moved to Fig. 5. No new material was added but a bit of repetition was removed.

Fig. 3 AB are still not well explained for the general audience (legends).

"Results of DFT calculations for packing of the various dimers within the unit cell parameters. Polar plots represent dihedral angles for vinyl groups attached to the respective pyrrole rings (??? What are the axes?): B,C for the upper heme, and B′,C′ for the lower heme."

Panels A and B now appear as D and E in the same figure, to match the order of presentation in the text. Small stick diagrams were added in order to clarify the meaning of the dihedral angle shown in the polar graph plots, and the legend was updated accordingly. We believe that the interpretation is much more intuitive now.

Additional Questions:

Quality of experimental data, technical rigor: Moderate

Significance to chemistry researchers in this and related fields: Moderate

Broad interest to other researchers: Moderate

Novelty: High

Is this research study suitable for media coverage or a First Reactions (a News & Views piece in the journal)?: No

Reviewer: 3

Recommendation: Publish in ACS Central Science without change.

Comments:

I thank the authors for replying to my comments and depositing the kinematical refinement. I believe the manuscript is ready for publication without changes.

Additional Questions:

Quality of experimental data, technical rigor: Top 1%

Significance to chemistry researchers in this and related fields: Top 1%

Broad interest to other researchers: Top 5%

Novelty: Top 1%

Is this research study suitable for media coverage or a First Reactions (a News & Views piece in the journal)?: Yes
